# Determining effective dietary and ruminal fermentation parameters on live weight gain in diets formulated with selected agro-industrial by-products and their validation in performance fattening lambs

**DOI:** 10.1093/tas/txae166

**Published:** 2024-12-11

**Authors:** Seyed Morteza Vaghar Seyedin, Mohsen Mojtahedi, Hossein Naeimipour Younesi, Einar Vargas-Bello-Pérez

**Affiliations:** Department of Animal Science , Faculty of Agriculture, University of Birjand, Birjand 97175-331, Iran; Department of Animal Science , Faculty of Agriculture, University of Birjand, Birjand 97175-331, Iran; Department of Animal Science , Faculty of Agriculture, University of Birjand, Birjand 97175-331, Iran; Department of International Development, School of Agriculture, Policy and Development, University of Reading, Reading RG6 6EU, UK; Facultad de Zootecnia y Ecología, Universidad Autónoma de Chihuahua, Chihuahua 31453, México

**Keywords:** acceptability, blood metabolite, gas production, rumen ammonia, sustainability

## Abstract

The objective of this study was to determine the effects of dietary agro-industrial by-products (AIBP) with different amounts of metabolizable energy (ME) and crude protein (CP) on fermentation (96 h) and gas production (GP) kinetics in vitro, as well as acceptability, animal performance, digestibility, and blood parameters in lambs. The gas production technique (GPT) and fermentation characteristics were used in an in vitro trial. This experiment used diets with ME contents of 6.28, 7.53, and 9.62 MJ/kg, as well as levels 120, 140, and 160 g/kg CP. Principal component analysis (PCA) was used to select the most important dietary ingredient ruminal fermentation parameters. The in vitro results showed that increasing ME and CP content increased and decreased GP (*P* < 0.05), respectively. An increase in CP content caused an increase in ruminal ammonia nitrogen (NH_3_-N) concentration (*P* < 0.05) and an increase in ME caused a decrease in pH (*P* < 0.05). PCA, by reducing the dimensions of the variables, shows that neutral detergent fiber (NDF), synchronization index (SI), and lignin as dietary parameters and pH and NH_3_ as fermentation quality parameters were the main variables in predicting LWG (*P* < 0.05). Next, to prove the ineffectiveness of dietary protein content on LWG, the highest ME content (ME = 9.62 MJ/kg) was selected for in vivo experiment. For the in vivo trial, eighteen male lambs of 6-7 months of age and 30.6 ± 2.27 kg body weight were randomly fed on three diets containing 120, 140, and 160 g/kg CP and ME 9.62 MJ/kg. There was no effect of the experimental diets on acceptability, performance characteristics, or apparent digestibility in an in vivo trial (*P* > 0.05). NH_3_-N concentration was affected by the varying content of CP (*P* < 0.05). The results of this study indicate that Khorasan Kurdish lambs have a high potential for using AIBP in their diet. Additionally, lamb producers can prevent N wastage without worrying about the loss of animal performance by using 120 g/kg CP, which meets the animals’ needs and has beneficial environmental consequences.

## Teaser

The increase in metabolizable energy (ME) and crude protein (CP) content caused an increase and a decrease in gas production, respectively. Additionally, as predicted by the principal component analysis, the use of 120 g/kg of CP with an ME content of 9.62 MJ/kg, while meeting the needs of fattening lambs, prevented N loss. Furthermore, the average final weight, daily weight gain, and feed intake of lambs fed levels of 12%, 14%, and 16% CP were the same.

## Introduction

Establishing food security as one of the main necessities of every country depends on prioritizing and improving the role of agriculture and animal husbandry as the main providers of food. The role of animal husbandry holds great importance in various agricultural sectors worldwide. But the global warming and climate change caused by humans (i.e., use of fossil fuels and animal husbandry), and natural activities (i.e., wetlands and oceans) (Vaghar Seyedin et al. 2022) have led to a decrease in suitable pastures for livestock grazing, a lack of arable land, a reduction in vegetation, water shortages, and as a result, insufficient suitable feeds for livestock production (Godde et al. 2021). The world’s population is constantly increasing, and it is predicted that animal production will increase by 57% to meet food needs by 2050 (Alexandratos and Bruinsma, 2012). Unfortunately, more than 820 million people were suffering from hunger in 2020, and almost 2 billion people worldwide were having food insecurity (Salem et al. 2021). In such circumstances, the use of native feed, agro-industrial by-products (AIBP), and wastes, as well as unconventional food items, is inevitable.

The digestion and transformation of AIBP, which are not usable for humans, into valuable products such as meat and milk is a characteristic unique to ruminants, especially sheep. This trait has made sheep breeding economically important. However, providing the optimal level of required metabolizable energy (ME) and crude protein (CP) is a crucial topic for animal nutritionists, as it affects rumen growth and development, rumen fermentation parameters, amino acid absorption, and ultimately livestock performance at different breeding ages (Mi et al. 2022). Increasing the content of ME and CP in the diet raises blood glucose and urea nitrogen, respectively (Saro et al. 2020; Vastolo et al. 2022).

Except for common crops, such as wheat, rice, barley, corn, and sugarcane, there is a significant area dedicated to the cultivation of strategic products, such as pistachios (*Pistacia vera*), pomegranates (*Punica granatum*), barberry (*Berberis vulgaris*), and saffron (*Crocus sativus*) in Iran. However, the waste disposal of these products after processing, either in landfills or through incinerators, poses environmental challenges, including soil, air, and water pollution (Ghavipanje et al. 2016). By turning valueless raw materials into high-quality and affordable animal feeds that do not compete with human food, the use of AIBP in animal feed presents an opportunity to lower imports, minimize food waste, and create a clean and circular economy. In the meanwhile, tropical plants like *Berberis vulgaris* and *Alhagi* have been shown to have substantial levels of phenolic compounds and tannins, which have beneficial effects on animal health and rumen fermentation (Vaghar Seyedin et al. 2023).

The potential of using these by-products in ruminant diets has been established in many studies. For instance, Vaghar Seyedin et al. (2023) and Ghavipanje et al. (2016) observed an increase in feed consumption by adding *berberis vulgaris leaf* (BVL) to the lambs and goats ration, which indicates the acceptability of this AIBP. However, prior research demonstrates that high levels of plant secondary metabolites in AIBP might negatively impact the diet’s acceptability (Terranova et al. 2020). Also, by substituting 150 g/kg of BVL with alfalfa, Vaghar Seyedin et al. (2022) found an increase in glutamic pyruvic transaminase content and a decrease in blood glucose levels.

Most studies have only investigated the inclusion of one AIBP, and the effect of multiple byproducts together on rumen fermentation is unclear. Also, due to the large volume of AIBP produced in developing countries, there is a good potential to use these by-products as much as possible to preserve the environment. This study was performed to formulate rations based on AIBP at different levels of ME and CP and investigate their effects on fermentation and gas production (GP) kinetics in vitro as well as acceptability, animal performance, digestibility, and blood parameters in vivo.

## Materials and methods

### Ethical Considerations and Study Site

This experiment was carried out in two parts, in vitro and in vivo, respectively, in the animal nutrition laboratory and research farm of the Faculty of Agriculture, Birjand University, Iran. The farm’s position was 1,491 m above sea level, and it received 171 mm of rain on average each year. Furthermore, the average annual minimum and maximum temperatures were 8 and 24 °C. Keeping and caring for the experimental animals were also performed following the instructions of the Iranian Animal Care Council (IACC) regarding the protection of animals (Protocol no. 19293). The study was performed according to the guidelines of the Regulation on the Animal Care and Use Committee of Birjand University under the approved project ID 4001814.

### In Vitro Trial

#### Preparation and chemical analysis.

Samples were prepared by grinding and passing them through a sieve with a pore diameter of 1 mm. For this purpose, the recommended methods of AOAC (2012) were used. Measurements were taken of dry matter (DM; method 934.01), ash (method 942.05), CP (method 981.10), and ether extract (EE; method 920.29). Moreover, the neutral and acid detergent fiber (NDF and ADF) of the ingredients were assessed using the methods suggested by Van Soest et al. (1991).

### Ration Software Balance

The ideal levels of dietary ME and CP were tested using a 3 × 3 factorial experiment with the by-products and agricultural waste created in the preceding phase. The factors used in this experiment included dietary ME at three levels 6.28, 7.53, and 9.62 MJ/kg DM (ME6.28, ME7.53, and ME9.62) and CP at three levels 120, 140, and 160 g/kg DM (CP120, CP140, and CP160). Diets were balanced according to the recommendations of the National Research Council (NRC, 2007) and using Small Ruminant Nation System (SRNS) software version 1.9 (Table 1). In formulating the experimental rations, the percentage of food items was adjusted to have the least changes at each level of ME and CP.

**Table 1.** Ingredient and chemical composition of experimental diets

Item	LE			ME			HE		
Feedstuffs (g/kg)	LP	MP	HP	LP	MP	HP	LP	MP	HP
Alfalfa hay	-	-	-	100	100	100	80	80	80
*Berberis vulgaris leaf*	-	-	-	150	150	150	120	120	120
Sugarcane bagasse	300	300	300	250	250	250	-	-	-
Corn silage	-	-	-	-	-	-	80	80	80
Alhagi	400	400	400	-	-	-	120	120	120
Corn grain	40	40	40	160	160	160	250	250	250
Barley grain	45	45	45	115	115	115	150	150	150
Beet pulp sugar	20	20	20	40	40	40	60	60	60
Wheat bran	90	90	90	90	90	90	90	90	90
Meat meal	20	45	70	-	20	40	-	6	11
Safflower meal	-	-	-	70	45	20	25	12	-
Poultry litter	60	30	-	-	-	-	-	-	-
Urea	-	5	10	-	5	10	-	7	14
salt	5	5	5	5	5	5	5	5	5
Calcium carbonate	3	3	3	3	3	3	3	3	3
Sodium bicarbonate	7	7	7	7	7	7	7	7	7
Vitamin-premix	10	10	10	10	10	10	10	10	10
Chemical composition									
Metabolism energy (MJ/kg)	6.28	6.28	6.28	7.53	7.53	7.53	9.62	9.62	9.62
Crude protein (g/kg)	120	140	161	121	141	160	121	141	161
RDP (g/kg)	281	342	378	366	372	460	462	478	500
RUP (g/kg)	719	658	622	634	628	540	538	522	500
Crude fat (g/kg)	24	26	28	30	29	29	30	29	28
Ash (g/kg)	113	113	113	82	85	88	86	87	87
Non-fiber carbohydrate (g/kg)	263	263	260	360	354	349	480	477	474
Neutral detergent fiber (g/kg)	508	496	487	415	408	401	312	308	305
Lignin (% NDF)	23.95	24.37	24.67	28.21	28.21	28.71	23.38	22.70	23.05
LWG predict (g/d)	50	48	45	89	84	82	159	158	154
Synchronization index	0.73	0.71	0.69	0.76	0.78	0.75	0.74	0.66	0.63

Abbreviations: LE, low metabolizable energy; HE, high metabolizable energy; LP, low crude protein; MP, medium crude protein; HP, high crude protein; RDP, Rumen degradable protein; RUP, Rumen undegradable protein; NDF, Neutral detergent fiber. Vitamin-premix containing vitamin A (250,000 IU/kg), vitamin D (50,000 IU/kg) and vitamin E (15,000 IU/kg), calcium (120,000 mg/kg), magnesium (20,500 mg/kg), phosphorus (20,000 mg/kg), sodium (18,600 mg/kg), iron (12,500 mg/kg), copper (12,500 mg/kg), zinc (7,700 mg/kg), sulfur (3,000 mg/kg), manganese (2,250 mg/kg), iodine (56 mg/kg), cobalt (14 mg/kg) and selenium (10 mg/kg).

### Ruminal Kinetics and Synchrony Index

The ruminal degradation kinetics was determined using the nylon bag method (Ørskov & McDonald, 1979). Three non-lactating Holstein cows with rumen fistula (body weight 724 ± 48 kg and age 5 ± 1 years) were used on two separate runs and 200 g of each ration were weighed and placed in labeled polyester bags (10 × 20 cm dimensions and 40 to 55 µm pore diameter). The openings of the bags were sealed with a plastic thread. Incubation was done at 2, 4, 6, 8, 12, 16, and 24 h. After the incubation time, the bags were washed with cold water until clear water flowed out of the bags. The bags were dried in a forced air oven (Oven, 18273, Behdad Medical Production Company, Tehran, Iran) at 65 °C for 48 h. Then, the contents of the bags were used to measure the amount of ash and nitrogen. After removing the bags from the rumen, all the bags were washed with cold water for half an hour. It should be noted that zero-time bags were not placed in the rumen and were washed only with cold water (rapid fraction). Kinetic parameters of nutrient disappearance were estimated using Orskov and McDonald (1979). Next, the synchrony index of energy and protein of experimental diets was calculated based on the quantity of degradation of OM and CP (hourly) based on the suggested formula of Sinclair et al. (1995):


$$\begin{array}{l} SI=\;1\frac{{-}^{1}{/}_{n}\overset{n}{\underset{1}{\sum }}\sqrt{{\left(25-1000\;\left(\frac{CP}{OM}\right)\right)}^{2}}}{25} \end{array}$$

Where synchronization index (SI) is synchrony index, n is number of observations, the coefficient of 1,000 is for converting the unit (g to kg), and the value of 25 indicates the maximum efficiency of microbial protein synthesis in the rumen, which can be assumed in the ratio of 25 (g/kg) of CP/OM.

### In Vitro Gas Production

GP test for adjusted rations was conducted using the procedure described by Blümmel et al. (1997), which involved measuring the GP potential (b), GP rate (c), and lag phase (λ). At the Birjand semi-industrial slaughterhouse, rumen fluid was collected from sheep that had been slaughtered [42.5 ± 1.3 body weight (BW); mean ± SD]. The liquid was then put into a flask after being filtered via four layers of gauze. The rumen fluid was immediately transferred to the laboratory under anaerobic conditions. Next, a homogeneous solution was prepared by mixing the rumen fluid and McDougall’s solution in a ratio of 2:1. Then, 50 mL of the solution was added to 120 mL bottles containing 500 mg of the experimental rations that had been previously weighed. These bottles were then placed in a water bath with a temperature of 38.6 ± 0.1 °C and monitored for GP at 2, 4, 6, 8, 12, 24, 36, 48, 72, and 96 h of incubation using a sphygmomanometer (model CPG 2400, Mensor). Gas pressure was converted to volume using the volume equation under standard pressure and temperature conditions (1 atm and 0 °C) as described by Blümmel et al. (1997). The experiment was conducted in three runs with eight repetitions (four vials for GP and four vials for fermentation parameters) in each run. Three blank glasses were used to correct the gas produced in the incubation conditions. The b, c, and λ parameters were calculated using the equation proposed by Ørskov and McDonald (1979):


$$\begin{array}{l} p(t)=\;b\;(1\;-\;exp^{\left[-c\left(time-\lambda \right)\right)]}) \end{array}$$

Additionally, two bottles from each treatment were withdrawn to evaluate the pH of the test rations 12 and 24 h after incubation. The pH readings were then recorded using a Metrohm 827 digital pH meter (Metrohm 827, Switzerland). Gravimetric analysis was used to calculate IVDMD, considering the initial DM weight and the final weights after 12 and 24 h of incubation. NH_3_-N concentration was also measured at these times using the colorimetry method (Broderick and Kang 1980).

### In Vivo Trial

#### Animals and diets.

Based on the findings of the first experiment, the total price of the ration, the behavior of the test rations concerning changes in fermentation and in vitro gas production (IVGP) parameters, and the ME level of 9.62 MJ/kg along with three CP levels (CP120, CP140, and CP160) were selected for the experiment on lambs. For the trial, 18 Khorasan Kurdish lambs were used, ranging in age from 6-7 months and weighing an average of 30.60 ± 2.27 kg [mean ± SD]. The experimental lambs were split into three groups of 6 lambs each at random, with each group receiving the chosen diets. The lambs were vaccinated against enterotoxemia (Polivac-CL, Turkey), and were given internal (Albazen 2.5%, Iran), and external (Iverject, Iran) antiparasitic drugs. The duration of this experiment was 84 d with 14 d of adaptation. The lambs had free access to clean water and were fed throughout the day. They fattened in individual stalls (1.5 m × 2 m) with a cement bed under natural light conditions during the experimental period.

#### Sampling and measurements.

On the first day of testing, the acceptability of the selected rations from the previous stage was determined using the short-term intake rate (STIR) method (Fonseca et al. 2012) with a slight modification. To accomplish this, nine lambs were randomly selected and given a 25% daily ration (400 g DM) for 60 min. Subsequently, the lambs were starved for 4 h and then fed 50% ration (800 g DM) again for 5 min. Then the remaining 25% (400 g DM) was given to the animals for 5 min. After this period, the residual feed was collected and weighed. Additionally, each animal was starved for another 30 min to determine the acceptability of another ration.

Each lamb’s daily feed intake was noted, and the next morning the feed residue was gathered, weighed, and recorded. Additionally, the weights of lambs were recorded fortnightly. In the final week of the trial, the apparent digestibility of three lambs from each group was evaluated using the acid insoluble ash (AIA) approach for 3 consecutive days of three lambs from each group (Van Keulen and Young 1977). A stomach tube attached to an Erlenmeyer flask connected to a vacuum pump was used to collect rumen fluid from 3 lambs of each experimental group on days 14, 56, and 84 (Vaghar Seyedin et al. 2022). Filtrated rumen fluid was measured immediately with a portable pH meter (Metrohm 827; pH Lab, Herisau, Switzerland). NH_3_-N concentration was determined by the procedure of (Broderick and Kang 1980).

After morning feeding, 10 mL of blood was collected from the jugular artery and transferred to test tubes containing 0.1 mL of 10% EDTA. Following centrifugation (3,000 RPM for 10 min), the blood samples were stored in a freezer (−21 °C) until the measurement of blood metabolites. Using a commercial kit from Pars Azmoun (manufactured in Iran), the concentration of blood metabolites such as glucose, cholesterol, blood urea nitrogen (BUN), and triglycerides was measured and examined using an autoanalyzer (Chem Gesan 200, Italy).

### Statistical Analysis

In vitro data were statistically analyzed based on a factorial completely randomized design by proc NLIN (for IVGP data) and proc GLM (for fermentation parameters) of Statistical Analysis System software version 9.4 (SAS/STAT, SAS Institute Inc., Cary, NC).


$$\begin{array}{l} Y_{ijk}=\;\mu \;+E_{i}+\;P_{j}+\;(E_{i}\times P_{j})\;+\;e_{ijk} \end{array}$$

Y_ijk_: the dependent variable; μ: the overall mean; E_i_: the ME level effect (6.28, 7.53, and 9.62 MJ/kg of DM); P_j_: crude protein level (12%, 14%, and 16% of DM), E_i_ × P_j_: interaction effect between ME and crude protein levels; and e_ijk_: the experimental error.

Correlation analysis between fermentation parameters and chemical compounds of diet was used to check the existence of a correlation relationship by proc CORR of SAS 9.4. In this research, multivariate principal component analysis (PCA) was used to evaluate variables affecting LWG and reduce the number of variables by proc PRINCOMP of SAS 9.4. PCA was performed on the data obtained from IVGP kinetics and fermentation parameters and the correlation matrix was calculated. By using the correlation matrix, the principal components with a larger Eigenvalues ≥1 selection and then the most effective features of each component based on specific coefficients above 0.5 were selected to study their relationship with predicting live weight gain (LWG). To determine the relationship between the principal components in this study, univariate regression analysis was performed. The significance of all explanatory coefficients was measured at the level of 5 and 1%.

As well as in vivo data were analyzed based on a completely randomized design by proc MIXED for repeated measurements.


$$\begin{array}{l} Y_{ijkl}=\mu +T_{i}+A_{j(i)}+W_{k}+T_{i}\times W_{k}+e_{ijkl} \end{array}$$

Y_ijkl_: the dependent variable; μ: the overall mean; T_i_: the fixed effect of diets; A_j(i)_: the random effect of j^th^ lamb within i^th^ diet; W_k_: the fixed effect of repeated measurements; T_i_ × W_k_: the interaction fixed effect between T_i_ and W_k_; e_ijkl_: experimental error.

Least-square means were computed and tested by Tukey’s test at a significant level of 0.05 for differences.

## Results

### In Vitro Experiment

#### IVGP kinetics.

**Figure 1.**
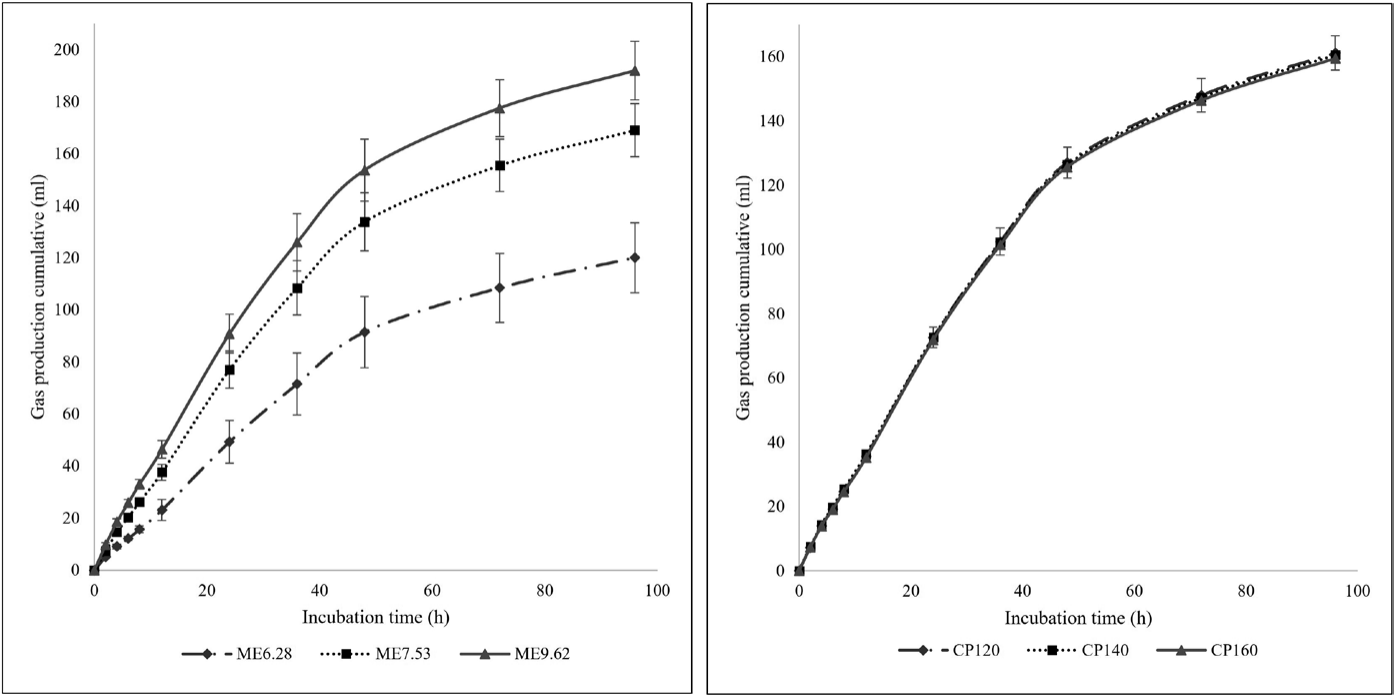
Effect of ME and CP content on cumulative gas.

The results of the GP test are shown in Table 2 and Figure 1. Different levels of ME and CP were found to have significant effects on the b and λ (*P <* 0.001). Specifically, the b value increased with increasing ME and decreased with increasing CP content. However, the same cumulative gas was observed at different levels of CP. Moreover, the c value increased significantly with a higher energy level (*P <* 0.001), but different levels of CP did not affect it (*P *= 0.859). Meanwhile, λ decreased as ME content increased (*P <* 0.001). The interaction effect of ME and CP on gas IVGP parameters was also significant (*P <* 0.05), with the highest and lowest b value observed at ME6.28 and CP160 and ME9.62 and CP160, respectively. Additionally, the c value at ME6.28 showed a decreasing trend compared to other levels of ME (*P <* 0.001). Finally, the lowest λ value was observed at ME9.62 and CP140 (*P <* 0.001).

**Table 2.** in vitro rumen gas kinetics (mL gas/g DM) of experimental diets

Main effects		Gas production parameters		
Source	level	b	c	λ
ME (MJ/kg)	6.28	147.11^c^	0.0192^c^	1.224^a^
	7.53	193.02^b^	0.0232^b^	1.110^b^
	9.62	215.61^a^	0.0245^a^	0.734^c^
CP (g/kg)	120	186.32^a^	0.0223	1.097^a^
	140	185.12^ab^	0.0224	0.934^b^
	160	184.30^b^	0.0222	1.038^ab^
SEM		0.3714	0.0001	0.0326
**Interaction effects**				
ME level	CP level			
6.28	120	148.98^c^	0.0193^d^	1.339^a^
6.28	140	147.68^c^	0.0192^d^	1.186^ab^
6.28	160	144.65^d^	0.0191^d^	1.147^ab^
7.53	120	193.86^b^	0.0232^c^	1.189^ab^
7.53	140	193.71^b^	0.0233^bc^	1.045^bc^
7.53	160	191.50^b^	0.0232^c^	1.097^abc^
9.62	120	216.11^a^	0.0245^a^	0.762^de^
9.62	140	213.98^a^	0.0246^a^	0.571^e^
9.62	160	216.74^a^	0.0243^ab^	0.869^cd^
SEM		0.6433	0.0002	0.0565
***P*-value**				
ME		0.001	0.001	0.001
CP		0.001	0.859	0.002
ME × CP		0.001	0.988	0.024

Abbreviations: LE, low metabolizable energy; HE, high metabolizable energy; LP, low crude protein; MP, medium crude protein; HP, high crude protein; b, gas production potential; c, gas production rate; λ, lag phase; SEM, standard error of means. Means are considered significantly different at P *< *0.05.

#### Ruminal fermentation parameters.

Table 3 shows the results regarding rumen fermentation parameters. The data indicates that an increase in energy content led to a decrease in pH and NH_3_-N concentration at 12 and 24 h after incubation (*P <* 0.001). Additionally, NH_3_-N concentration significantly increased at CP160 compared to CP120 and CP140 (*P <* 0.001). Conversely, IVDMD at 12 and 24 h were directly proportional to the ME content (*P <* 0.001), but unaffected by different CP contents (*P *= 0.686). Furthermore, significant interaction effects were observed for ME and CP content in pH, NH_3_-N concentration and IVDMD were also significant (*P <* 0.001). Hence, in 24 h, the lowest pH and NH_3_-N concentration were observed at ME9.62 and CP120, while the highest NH_3_-N concentration and pH were observed at ME6.28 and CP160 level and ME628 and CP120 levels, respectively (*P <* 0.001). However, IVDMD increased significantly after 24 h of incubation at ME9.62 compared to the groups with ME6.28 and ME7.53 groups.

**Table 3.** In vitro ruminal fermentation parameters

Main effects		Ruminal Fermentation Parameters					
Source	level	pH		NH_3_-N		IVDMD	
		12	24>	12	24	12	24
ME (MJ/kg)	6.28	6.91^a^	6.34^a^	6.04^a^	11.97^a^	22.65^c^	33.63^c^
	7.53	6.67^b^	6.25^b^	5.57^b^	10.95^b^	28.74^b^	44.59^b^
	9.62	6.54^c^	6.18^c^	5.20^c^	10.16^c^	32.45^a^	53.72^a^
CP (g/kg)	120	6.66^c^	6.24	4.42^c^	9.47^c^	26.99	44.00
	140	6.71^b^	6.25	6.04^b^	10.55^b^	28.23	43.71
	160	6.76^a^	6.26	6.57^a^	13.06^a^	28.61	44.23
SEM		0.001	0.004	0.058	0.211	0.493	0.911
**Interaction effects**							
ME level	CP level						
6.28	120	6.95^a^	6.38^a^	5.15^d^	9.63^cd^	21.74^c^	32.99^c^
6.28	140	6.92^a^	6.33^b^	6.28^ab^	10.94^bcd^	22.78^c^	33.71^c^
6.28	160	6.87^a^	6.30^bc^	6.70^a^	15.33^a^	23.43^c^	34.20^c^
7.53	120	6.56^c^	6.20^ef^	4.04^e^	9.47^cd^	27.69^b^	44.33^b^
7.53	140	6.71^b^	6.24^de^	6.07^bc^	10.71^cd^	29.07^ab^	45.17^b^
7.53	160	6.75^b^	6.27^cd^	6.61^a^	12.67^b^	29.44^ab^	45.27^b^
9.62	120	6.46^d^	6.15^g^	3.41^f^	9.32^d^	31.56^ab^	54.68^a^
9.62	140	6.50^cd^	6.17^fg^	5.78^c^	9.99^cd^	32.83^a^	53.25^a^
9.62	160	6.66^b^	6.21^e^	6.40^ab^	11.17^bc^	32.94^a^	53.23^a^
SEM		0.019	0.007	0.101	0.366	0.853	1.578
***P*-value**							
ME		0.001	0.001	0.001	0.001	0.001	0.001
CP		0.001	0.050	0.001	0.001	0.079	0.686
ME × CP		0.001	0.001	0.001	0.001	0.001	0.001

Abbreviations: LE, low metabolizable energy; HE, high metabolizable energy; LP, low crude protein; MP, medium crude protein; HP, high crude protein; NH_3_-N, ammonia nitrogen concentration; IVDMD, in vitro dry matter disappearance; SEM, standard error of means. Means are considered significantly different at *P* *< *0.05.

#### Correlation and principal component analysis.

Positive correlations were observed between LWG × ME, RDP, NFC, and GP24 with *r* = 1.00, 0.83, 0.99 and 0.86, and 0.94, respectively (*P <* 0.05), and between IVDMD × ME, RDP, NFC and GP24 with *r* = 0.95, 0.85, 0.96, and 0.88, respectively, whereas Negative correlations of high magnitude were observed between LWG × neutral detergent fiber (NDF), RUP, pH, and ash with *r* = −0.98, −0.87, −0.83, −0.87, and −0,73, respectively (*P <* 0.05; Table 4).

**Table 4.** Pearson partial correlation coefficients for rations chemical composition and rumen fermentation parameters of the full data set

	ME	Ash	SI	RDP	RUP	NFC	NDF	Lignin	GP24	IVDMD	pH	NH3	LWG
ME	1												
Ash	−0.75	1											
SI	−0.39	−0.16	1										
RDP	0.86	−0.65	−0.47	1									
RUP	−0.86	0.65	0.47	−1.00	1								
NFC	1.00	−0.79	−0.34	0.85	−0.85	1							
NDF	−0.99	0.81	0.34	−0.90	0.90	−0.99	1						
Lignin	−0.37	−0.33	0.76	−0.20	0.20	−0.31	0.25	1					
GP24	0.87	−0.84	−0.14	0.75	−0.75	0.88	−0.89	−0.05	1				
IVDMD	0.95	−0.83	−0.23	0.85	−0.85	0.96	−0.97	−0.17	0.88	1			
pH	−0.88	0.86	0.11	−0.77	0.77	−0.90	0.90	0.06	−0.84	−0.90	1		
NH3	−0.39	0.39	−0.20	0.05	−0.05	−0.41	0.33	0.13	−0.39	−0.37	0.33	1	
LWG	1.00	−0.73	−0.40	0.83	−0.83	0.99	−0.98	−0.40	0.86	0.94	−0.87	−0.42	1

Abbreviations: ME, metabolizable energy; SI, synchronization index; RDP, rumen degradable protein; RUP, rumen undegradable protein; NFC, non-fiber carbohydrate; NDF, neutral detergent fiber; GP24, gas production at 24h incubation; IVDMD, in vitro dry matter disappearance; NH_3_, ammonia; LWG, live weight gain.

**Figure 2.**
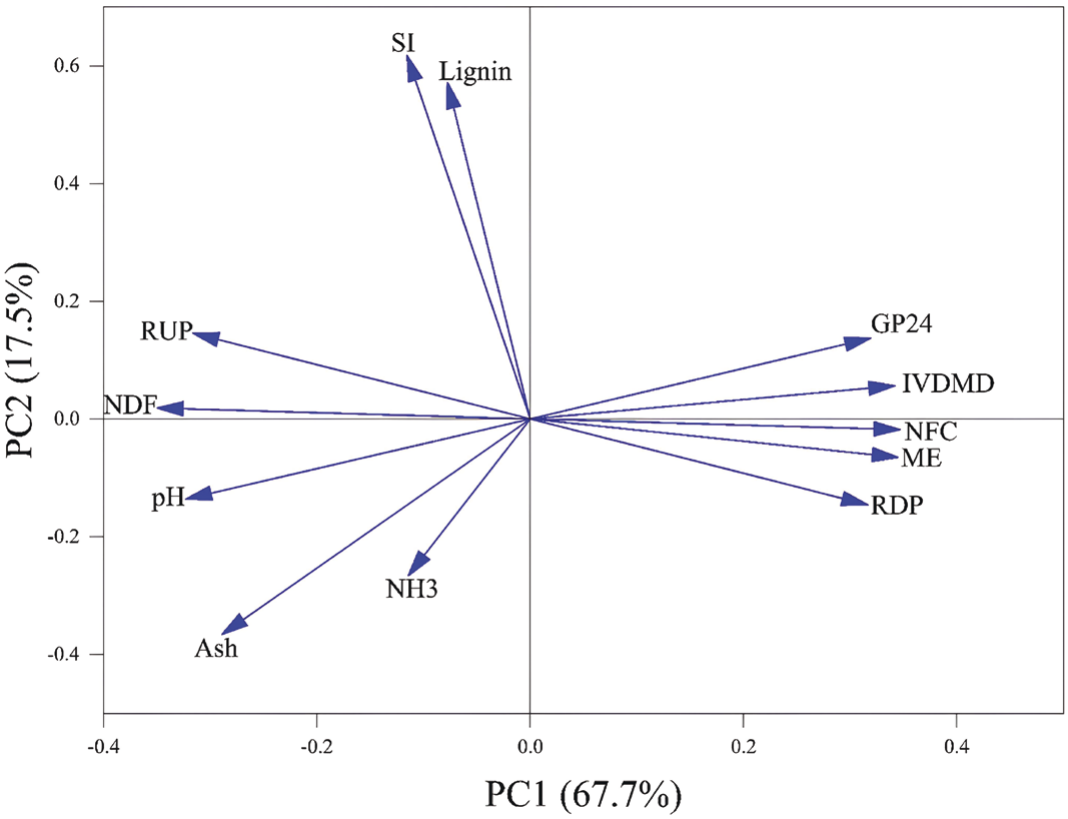
PCA Correlation of 12 characteristics of chemical composition and in vitro ruminal fermentation as a function of the first four components.

In Figure 2, the correlation is presented as a schematic of the coefficients and the degree of correlation of the variables with PC1 and PC2 of PCA. The vectors show the value of the coefficients (correlation) of the variables with PC1 (x-axis) and PC2 (y-axis).

CP was eliminated as this was not found to be correlated (*P* > 0.05) with LWG (data not shown). However, ME, Ash, SI, RDP, RUP, NFC, NDF, lignin, GP24, IVDMD, pH, and NH_3_ were found to be correlated with LWG (Table 4). PCA results show the first four principal components accounted for approximately 97% of the total variation. The PCA (Table 5 and Figure 2) plotted considering LWG as the main attribute revealed that the PC1 had relatively more influence (67.7%) than the PC2 (17.5%). The first PC explained 68% of the total variation and absolute varimax rotated factor loadings of NDF, NFC, and ME, IVDMD had the highest weighing factors. However, NFC, ME, and IVDMD were found to be correlated with NDF (*r* > 0.95). Thus, only NDF from the first PC was chosen because it was the original variable having the highest rotated factor loading.

**Table 5.** Rotated factor loadings for the first four principal components (PCs) generated from original variables

Item	Principal components			
	PC1	PC2	PC3	PC4
ME	0.345	−0.065	−0.118	−0.090
Ash	−0.289	−0.366	−0.092	−0.197
SI	−0.115	0.617	0.007	−0.535
RDP	0.316	−0.146	0.310	0.010
RUP	−0.316	0.146	−0.310	−0.010
NFC	0.347	−0.018	−0.117	−0.083
NDF	−0.350	0.019	0.016	0.043
Lignin	−0.078	0.571	0.435	0.350
GP24	0.319	0.137	−0.028	0.468
IVDMD	0.342	0.056	−0.027	−0.113
pH	−0.323	−0.136	−0.011	0.498
NH_3_	−0.114	−0.266	0.761	−0.235
Eigenvalue	8.120	2.095	1.223	0.194
Percent (%)	67.67	17.46	10.19	1.62
Cumulative percent (%)	67.67	85.13	95.32	96.94

Abbreviations: ME, metabolizable energy; SI, synchronization index; RDP, rumen degradable protein; RUP, rumen undegradable protein; NFC, non-fiber carbohydrate; NDF, neutral detergent fiber; GP24, gas production at 24h incubation; IVDMD, in vitro dry matter disappearance; NH_3_, ammonia.

Concerning the second PC, SI, and lignin have the highest rotated factor loadings. However, SI and lignin were correlated with each other (*P* < 0.05), thus, SI was chosen as an important dietary ingredient parameter. Ten percent of the total variation explained by the third PC having the highest rotated factor loadings for NH_3_ was selected rotated factor loadings were within 10% of the highest factor loadings. From the fourth PC, the highest rotated factor loadings were found SI, pH, lignin, and GP24. Considering that SI was already selected and there was a high correlation between pH and GP24 (*r* > 0.80), lignin and pH (*P* = 0.51) were chosen, and respective rotated factor loadings were within 10 % of the highest factor loadings. Therefore, NDF, SI, lignin, pH, and NH_3_ were selected as the final 5 dietary ingredients and ruminal fermentation parameters for developing the new index. Then, the following equation was calculated according to the regression of NDF, SI, lignin, pH, and NH_3_ with LWG:


$$\begin{array}{lll} LWG=\; & 790.31-0.47\;NDF+104.92\;SI-4.72\;Lignin\; & -70.67\;pH-1.83\;NH_{3} \end{array}$$

### In Vivo Experiment

#### Animal performance and digestibility.

The STIR, performance characteristics, and appearance digestibility of lambs fed with experimental diets are shown in Table 6. None of the STIR and performance characteristics were affected by the experimental diets (*P *= 0.751). Additionally, the apparent digestibility was the same between all groups receiving experimental diets (*P *> 0.05).

**Table 6.** Nutrient digestibility, growth performance, and feeding cost in fattening lambs fed experimental diets

Items	Experimental treatments			SEM	*P*-value
	LP	MP	HP		
STIR (g/min)					
400 g Intake in 50 min	7.62	7.76	7.62	0.102	0.519
800 g Intake in 5 min	48.77	49.77	45.55	1.657	0.191
400 g Intake in 5 min	23.66	22.00	24.33	2.333	0.769
Total intake in 60 min	14.27	14.21	14.49	0.270	0.751
Performance					
Initial weight (kg)	27.83	27.45	28.13	0.979	0.886
Final weight (kg)	50.78	51.40	51.55	0.807	0.779
Average daily gain (g)	273.21	285.12	279.77	7.658	0.696
Feed intake (g/d)	1556.95	1523.94	1532.25	12.481	0.185
Feed conversion ratio	5.74	5.38	5.53	0.231	0.553
Digestibility (g/kg)					
DM	658.5	656.4	657.0	23.23	0.998
OM	702.6	698.4	701.2	20.62	0.989
CP	688.0	706.0	718.3	24.06	0.675
NDF	666.3	659.1	648.1	21.31	0.833
ADF	589.9	582.8	581.7	19.41	0.948

Experimental treatments: LP, MP and HP includes 120, 140 and 160 g/kg CP, respectively. Abbreviations: LP, low crude protein; MP, medium crude protein; HP, high crude protein; STIR, short term intake rate; DM, dry matter; OM, organic matter; CP, crude protein; NDF, neutral detergent fiber; ADF, acid detergent fiber; SEM, standard error of means. Means are considered significantly different at P *< *0.05.

#### Rumen fermentation.

**Figure 3.**
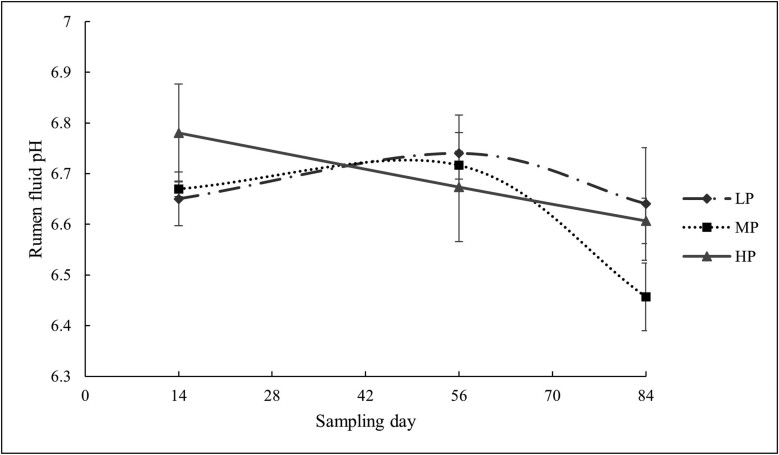
Effect of experimental treatments on lambs rumen fluid pH.

**Figure 4.**
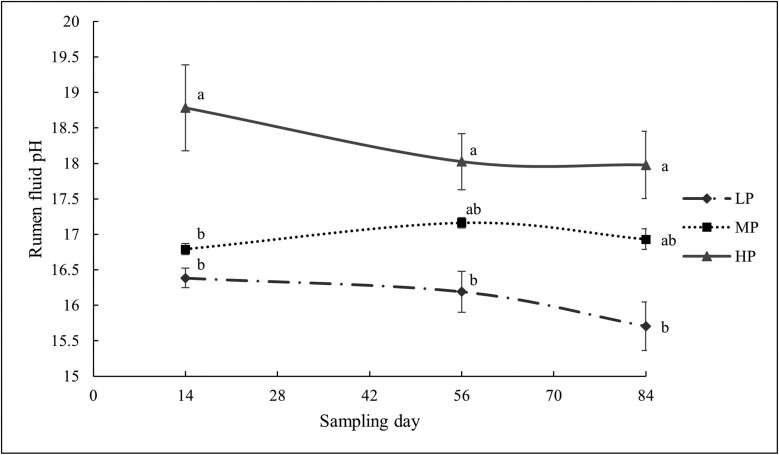
Effect of experimental treatments on lambs’ ammonia nitrogen (NH3-N) concentration.

The pH was not affected by CP content (*P *= 0.606, Figure 3). CP contents on the course of the trial and on different days influenced the NH_3_-N concentration. (*P *< 0.001). In comparison to the LP diet, the HP diet had the greatest NH_3_-N concentration on all sample days (*P *< 0.001, Figure 4). However, there was no statistically significant difference in NH_3_-N concentration between the LP and MP treatments (*P *= 0.103).

#### Blood indicators.

Regarding the blood indicator results, the glucose blood of the experimental lambs had no difference (*P* = 0.529, Table 7). The lambs fed the HP diet showed a higher total CP, albumin, and BUN compared with the LP group (*P <* 0.05); however, no differences were observed between the MP and HP groups.

**Table 7.** Serum biochemical indicators of lambs fed experimental diets

Serum blood indicators	Experimental treatments			SEM	*P*-value
	LP	MP	HP		
Glucose (mg/dL)	64.24	64.65	63.66	0.614	0.529
Total Protein (g/dL)	6.18	6.29	6.31	0.085	0.659
Albumin (g/dL)	3.68^b^	3.75^ab^	3.86^a^	0.029	0.014
Blood urea nitrogen (mg/dL)	15.75^b^	19.92^a^	20.92^a^	0.788	0.008
Cholesterol (mg/dL)	57.50	63.09	59.01	2.793	0.400
Triglyceride (mg/dL)	23.87	28.24	28.93	1.590	0.077

Experimental treatments: LP, MP and HP includes 120, 140 and 160 g/kg CP, respectively. Abbreviations: LP, low crude protein; MP, medium crude protein; HP, high crude protein; SEM, standard error of means. Means are considered significantly different at *P* *< *0.05.

## Discussion

### In Vitro Trial

#### IVGP kinetics.

An alternate method for figuring out the nutritional content of feed is IVGP. This is because measuring cumulative GP makes it simple to evaluate the pace and amount of degradation and rumen fermentation (Blümmel et al. 1997). IVGP provides a lot of benefits, including being quicker, cheaper, and more effective when dealing with many samples. The results show that increasing the CP content of the diet results in less IVGP, and conversely, increasing the ME content leads to more IVGP. The exact cause of gas reduction at the level of 160 g/kg CP is not clearly understood. However, the decrease in GP with the increase in dietary protein levels is likely due to the fermentation of nitrogenous compounds, the production of NH_3_, and the neutralization of acids at this level. This discovery could have an explanation due to the additional energy that the higher concentration made accessible to the microbial community. On the other hand, the reduction of NDF and ADF is another reason for the increase in GP (Niu et al. 2018; Wang et al. 2022). However, it should not be forgotten that the dietary CP also produces a part of GP, which is not a significant number compared to carbohydrates in the whole fermentation process. The maximum volume of GP is achieved by the simultaneous fermentation of several substrates. In such a process, it is challenging to relate each division to another factor because the substrates have different speeds in fermentation.

Malekkhahi et al. (2023) show that sugarcane molasses and sugar beet pulp are mainly used as an energy source in ruminant nutrition as well as to encourage feed intake. These substances also greatly boost GP and accelerate fermentation kinetics (Vastolo et al. 2022). Additionally, it has been noted that diets containing sugarcane molasses, which are primarily made up of soluble carbohydrates, have been shown to hasten the rate of fermentation in the rumen (Li et al. 2014). Likewise, Vaghar Seyedin et al. (2019) and Vastolo et al. (2022) noted that diets with higher NFC intake demonstrate higher IVGP and the findings of the current study support their observations. Also, the reduction of GP with the inclusion of by-products such as BVL, sugarcane bagasse, and Alhagi is likely due to the lower nutritional value of these materials compared to common forage like alfalfa hay (Kazemi and Bezdi, 2021; Vaghar Seyedin et al., 2023). By-products often contain less digestible fiber and protein, which can lead to decreased fermentation efficiency in the rumen. Consequently, this results in lower GP during incubation. Additionally, the presence of certain PSMs (tannins and polyphenolic compounds) in the BVL and Alhagi may influence microbial populations, thereby reducing GP (Kazemi and Bezdi, 2021; Vaghar Seyedin et al., 2023).

#### Ruminal fermentation parameters.

Ruminal fermentation indicators, such as NH_3_-N concentration and pH, prove the rumen ecosystem’s changes and circumstances throughout fermentation. The normal physiological pH range of the rumen is between 5.5 and 7.0 (Krause and Oetzel 2006). In the present study, the pH did not vary significantly across CP levels, which is consistent with previous studies (Van Dung et al. 2014). However, increasing the ME in the diet led to a reduction in pH value. The decrease in pH has a direct relationship with the increase in NFC of the diet and an inverse relationship with its NDF content. For example, LE had an NFC value of 260 g/kg, while HE had a value of 480 g/kg. The pH values in the present study remained relatively stable and were greater than the suggested range of 5.0 to 5.5 by Hoover (1986). This would not negatively affect rumen microbial activity (Prez et al., 2024).

Moreover, the optimal range of NH_3_-N as an N source for microbial protein synthesis is between zero and 50 mg/dL (Wang et al. 2022). Maintaining the NH_3_-N concentration within this limit is necessary for rumen fermentation. High NH_3_-N concentration indicates rapid decomposition of CP sources and lack of microbial protein synthesis, resulting in nitrogen loss in the rumen N cycle. On the other hand, low NH_3_-N limits the efficiency of cellulose-degrading bacteria (Hristov et al. 2002).

As anticipated, variations in CP content had an impact on the rumen’s fermentation patterns and NH_3_-N concentration (Mutsvangwa et al. 2016). A rise in the diet’s CP content raises the NH_3_-N concentration (Mutsvangwa et al. 2016), which suggests that microorganisms are consuming it in the form of NH_3_ (Mutsvangwa et al. 2016; Putri et al., 2021). The high level of soluble protein and IVDMD in the diet, on the other hand, is reflected in the high rumen fluid NH_3_-N concentration (Rosmalia et al. 2022). CP is made up of two fractions, rumen degradable protein (RDP) and rumen undegradable protein (RUP). The amount of RDP and the ratio of RDP to RUP play a crucial role in controlling the NH_3_-N concentration of rumen liquid. RDP is utilized by rumen microbes as a source of N for microbial protein synthesis, and the result of their activity is NH_3_ (Mutsvangwa et al. 2016; Putri et al. 2021). RDP is initially degraded and transformed into peptides by the production of protease enzymes by rumen microorganisms (mostly proteolytic). Peptides are then transformed into amino acids by the secretion of the enzyme peptidase. To create NH_3_, bacterial deaminase enzymes must be secreted (Alves et al. 2021). This results in microbial protein synthesis. The findings of the current study corroborate the findings of Putri et al. (2021), who found that increasing the CP content of the diet from 120 to 140 g/kg led to an increase of 7.73 mM in NH_3_-N production.

Vaghar Seyedin et al. (2024) reported that the NH_3_-N concentration of raw and processed poultry litter ranges from 4.73 to 18.36 (mg/dL). Most tropical plants, including BVL and Alhagi, have high concentrations of PSMs, including phenolic compounds and tannins (Kazemi and Bezdi 2021; Vaghar Seyedin et al. 2023). Utilizing plants that contain PSMs, particularly tannins has been shown to disrupt the rumen’s NH_3_-N. Wang et al. (2018), for instance, showed that consuming AIBPs including grapes, hazelnuts, and birch that contained more than 18 g/kg of tannin decreased the amount of NH_3_-N. However, rosebay willow, green grapevine, and silver birch leaf produced comparable results (Terranova et al., 2020). Tannin was present in substantial concentrations in all plants. Tannins in particular are well known for their capacity to bind with proteins to create insoluble complexes (Ghavipanje et al. 2016).

IVDMD increased with the increasing energy content, which is consistent with the results of Van Dung et al. (2014). The increased inclusion of concentrate in the diets may have contributed to the higher level of soluble substrates, which led to enhanced DM and OM digestibility. Also, Van Dung et al. (2014) reported that the highest digestibility was achieved under experimental conditions in a diet with a forage:concentrate (F:C) ratio of 20:80 compared to a F:C ratio of 80:20. These authors acknowledged that increasing the concentration of the diet (i.e., ME) can improve IVDMD. However, the results of Vaghar Seyedin et al. (2019, 2020) show that increasing the energy of the diet, after supplementing high content of carbohydrates, can reduce IVDMD by producing lactic acid. Additionally, the more degradable the feed, the more microorganisms in the rumen will be active. However, microorganisms are less active when IVGP is higher (Wang et al. 2022). In general, the buffer solutions used in the IVGP are selected in such a way that they provide enough buffering capacity during the incubation time (96 or 120 h) to maintain the pH and prevent its drop. In the present study, diets with different CP had the same IVDMD value. Our results were in agreement with the study reported by Mi et al. (2022), which found that dietary CP did not affect IVDM in the rumen. Additionally, our results were consistent with those of Van Dung et al. (2014), who reported that increasing the ME of the diet increased IVDMD but that CP160 and CP190 content did not have a significant effect on degradability.

##### Correlation and PCA

Until this experiment, no other studies had been found regarding the effects of SI, ME, CP content, GP, and fermentation parameters on LWG using the PCA. Therefore, other findings are discussed. In agreement with our results in the study of Tharangani et al., (2021), lignin (−0.61) and starch as a part of NFC content (0.85) were related to milk production. Lignin, SI, RUP, and NDF had a significant impact on LWG. The close angle of NDF and RUP on PC loadings revealed that they were closely correlated with LWG as compared to the SI and lignin. The IVDMD had more influence on PC1, whereas GP24 had an impact on PC2 and was found to be positively correlated with the LWG.

**Figure 5.**
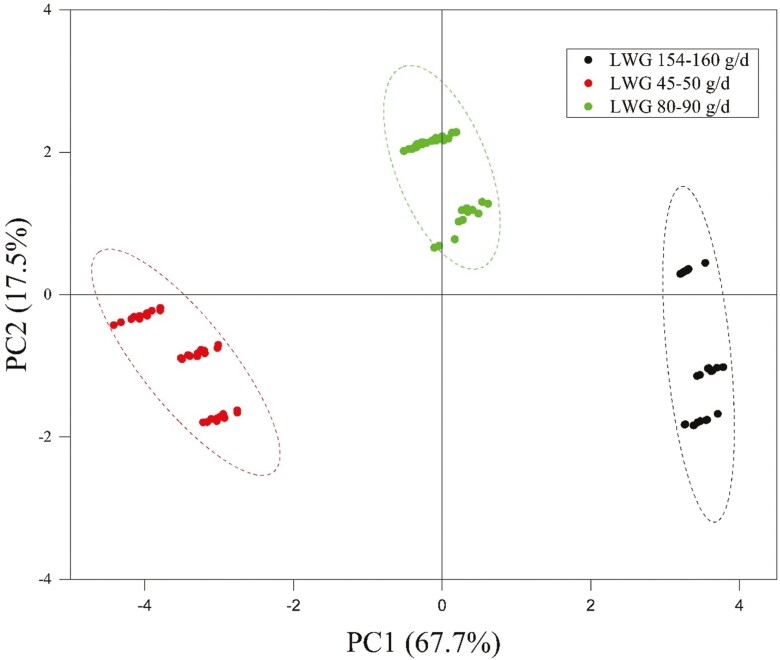
Principal component analysis (PCA) of dietary quality and ruminal fermentation parameters on LWG (live weight gain).

The PCA score plot of the dietary chemical composition and ruminal fermentation (Figure 5) showed that variables clustered separately between the three groups of LWG. In this research, five dietary quality parameters (i.e., NDF, SI, and lignin as nutritional quality parameters and pH and NH_3_ as fermentation quality parameters) were used to develop the final indexes. Each of these vectors are orthogonal to each other in multidimensional space. The length of the vectors indicates the intensity of the relationship and the angle between the two vectors indicates the degree of correlation. Additionally, the findings of a study on the use of PCA indicate that the inclusion of certain tropical plants containing phenolic compounds is effective in reducing CP degradability (Jayanegara et al., 2011). This significance may also influence the digestibility of some rations in this experiment that included BVL.

In general, the results of the first experiment showed that: 1) the ME, NFC, NDF, SI, RDP, GP, pH, NH_3,_ and IVDMD are the main variables in predicting LWG; and 2) the PCA method identified NDF, SI, lignin, pH and, NH_3_ as reliable variables for predicting LWG by reducing the dimensions of the variables.

### In Vivo Trial

#### STIR, characteristic performance, and digestibility.

It was found that the diet acceptability of the experimental rations was the same, based on the STIR method. The same STIR is likely caused by the components in the ration not having undergone any significant modifications. In this regard, our previous studies did not show any change in feed intake of lambs fed with zero and 7.5 g/kg of BVL (Vaghar Seyedin et al. 2022). DM intake is an important factor in lamb production, as it determines nutrient absorption for maintenance, growth, and body achievements (Saro et al. 2020; Vaghar Seyedin et al. 2023). Previous studies have linked feed consumption to the energy density of the diet and have reported a direct relationship between them (Devant et al. 2022). Furthermore, this parameter is less affected by the CP content of the diet (Saro et al. 2020). However, interactions between dietary CP content and feed intake in ruminant diets are often observed due to changes in rumen fermentation and microbiome (fibrolytic microorganisms), which can reduce fiber digestibility under N limitation (Saro et al. 2020). The present study did not observe any of the aforementioned cases.

Different content levels of dietary CP did not affect the total digestive tract digestibility of DM, OM, NDF, and ADF, which is consistent with the findings of Xia et al. (2018). These authors did not observe a change in the digestibility of nutrients in Holstein bulls when examining levels of 10%, 12%, and 14% CP (Xia et al. 2018). However, Bahrami-Yekdangi et al. (2016) reported that different dietary CP levels affect the digestibility of DM, OM, ADF, and NDF. Furthermore, Van Dung et al. (2014) demonstrated that different CP contents had varying effects on digestibility, with CP100, CP130, CP160, and CP190 leading to increased digestibility of CP but no changes in the digestibility of DM, OM, and NDF, which is in line with our results. Also, Devant et al. (2022) reported a decrease in the digestibility of OMD, DMD, and CPD in fed calves with levels of CP125 and CP170, which contradicts our results. Proper formulation of diets is crucial for enhancing digestibility and feed utilization as nutrient digestibility reflects microbial activity and fermentation in the rumen (Bahrami-Yekdangi et al. 2016). Nevertheless, the insignificant difference in nutrient digestibility among different experimental groups may be attributed to the lower variation of dietary CP in the current study. Furthermore, the similarity in the content of NDF and ADF can be considered as another potential reason for the comparable digestibility observed through the AIA method in the current study, as pointed out by Xia et al. (2018).

#### Rumen fermentation.

Recent research demonstrates that, in addition to dietary energy, one key factor influencing DM intake and the bioavailability of nutrients is the ratio of dietary fiber to moisture (Wang et al. 2022). Because dietary fiber promotes chewing, ruminating, salivation, preserving rumen buffering capacity, and enhancing rumen fermentation efficiency. A sufficient amount of dietary fiber can boost DM intake, which is intimately tied to how quickly nutrients break down in the body (Li et al. 2016). In this study, the amount of fiber in LP, MP, and HP diets were 31.2, 30.8, and 30.2 mg/g, respectively, and probably the fact that it is the same among the experimental treatments is the reason for the same fiber digestibility. Bahrami-Yekdangi et al. (2016) and Xia et al. (2018) have reported a high positive correlation between dietary CP content and NH_3_-N concentration, Saro et al. (2020) did not observe any changes in NH_3_-N concentration in Assaf lambs. However, the rumen fluid in the study of Saro et al. (2020) was taken after slaughter and an 8 h fasting, which can affect the concentration of NH_3_-N concentration.

#### Blood indicators.

The glucose concentration of lambs fed with different CP content was observed to have a similar range (CP120 = 62.44, CP140 = 64.65, and CP160 = 63.66 mg/dL). In agreement with the results of the present experiment, the content of 102, 123, and 142 g/kg CP did not change the blood glucose concentration of Holstein bulls (Xia et al. 2018). The probability that factors such as ME and DM intake are the same among the experimental groups explains the lack of changes in blood glucose concentration. Also, the total protein was the same across all experimental groups. This blood indicator was not found to have changed by Bahrami-Yekdangi et al. (2016), which is consistent with the results of the present experiment. The liver is the site of albumin synthesis, and all albumin is distributed in the blood, interstitial space, and cells (Soeters et al. 2019). Albumin is considered a negative acute-phase protein, and low serum albumin concentrations may be caused by and reflect an inflammatory state (Soeters et al. 2019). As observed by this study, Devant et al. (2022) also reported that an increase in CP content in the diet increased BUN. It is expected that an increase in BUN, consistent with the findings of Bahrami-Yekdangi et al. (2016) and Xia et al. (2018) is a result of the diet’s positive effect on the rumen’s NH_3_-N. Blood cholesterol levels have been shown to decrease in response to saponin (Mohammadabadi et al. 2023), while they remained unchanged in response to flavonoids (Vaghar Seyedin et al. 2022), which are phytochemical secondary metabolites found in *Conocarpus erectus* and BVL, respectively.

Generally, the data from the second experiment indicated that: 1) CP content of 120, 140, and 160 g/kg is ineffective for LWG in lambs; and 2) increasing the CP content only raises the cost of production and nitrogen loss (by increasing NH_3_-N production). In general, a protein level of 120 g/kg DM is recommended for lamb producers.

## Conclusion

Taken together, the IVGP has a direct relationship with the content of ME in the diet and an inverse relationship with CP level. NH_3_-N concentration and pH were different at various contents of ME and CP. Also, different sources of protein (plant- and animal-derived protein sources) were ineffective in the acceptability of the experimental diets. The relationship between nutrient intake and digestibility was optimal with CP120 and increasing dietary CP (to 160 g/kg) only N loss and increases the cost of fattening Kurdish lambs. Considering our findings, PCA accurately predicted the effects of ME and CP contents on the LWG, as well as the status of fermentation parameters and their relationships with each other. The balance of rations based on AIBPs while satisfying livestock requirements can significantly reduce environmental pollution.

## Data Availability

The datasets used and analyzed during the current study are available upon request.
